# Gold nanoparticles reduce inflammation in cerebral microvessels of mice with sepsis

**DOI:** 10.1186/s12951-021-00796-6

**Published:** 2021-02-19

**Authors:** Davide Di Bella, João P. S. Ferreira, Renee de Nazare O. Silva, Cinthya Echem, Aline Milan, Eliana H. Akamine, Maria H. Carvalho, Stephen F. Rodrigues

**Affiliations:** 1 Laboratory of Hypertension, Diabetes and Vascular Biology, Department of Pharmacology, Institute of Biomedical Sciences, University of Sao Paulo, Av. Prof. Lineu Prestes, 1524, ICB I, sala 205, 2º andar, Butanta, 05508-900 Sao Paulo, Brazil; 2 Laboratory of Vascular Nanopharmacology, Department of Pharmacology, Institute of Biomedical Sciences, University of Sao Paulo, Av. Prof. Lineu Prestes, 1524, ICB I, sala 319, 3º andar, Butanta, 05508-900 Sao Paulo, Brazil

**Keywords:** Gold nanoparticles, Septic encephalopathy, Mice, Cell adhesion molecules

## Abstract

**Background:**

Sepsis is an emergency medical condition that can lead to death and it is defined as a life-threatening organ dysfunction caused by immune dysregulation in response to an infection. It is considered the main killer in intensive care units. Sepsis associated-encephalopathy (SAE) is mostly caused by a sepsis-induced systemic inflammatory response. Studies report SAE in 14–63% of septic patients. Main SAE symptoms are not specific and usually include acute impairment of consciousness, delirium and/or coma, along with electroencephalogram (EEG) changes. For those who recover from sepsis and SAE, impaired cognitive function, mobility and quality of life are often observed months to years after hospital discharge, and there is no treatment available today to prevent that. Inflammation and oxidative stress are key players for the SAE pathophysiology. Gold nanoparticles have been demonstrated to own important anti-inflammatory properties. It was also reported 20 nm citrate-covered gold nanoparticles (cit-AuNP) reduce oxidative stress. In this context, we tested whether 20 nm cit-AuNP could alleviate the acute changes caused by sepsis in brain of mice, with focus on inflammation. Sepsis was induced in female C57BL/6 mice by cecal ligation and puncture (CLP), 20 nm cit-AuNP or saline were intravenously (IV) injected 2 h after induction of sepsis and experiments performed 6 h after induction. Intravital microscopy was used for leukocyte and platelet adhesion study in brain, blood brain barrier (BBB) permeability carried out by Evans blue assay, cytokines measured by ELISA and real time PCR, cell adhesion molecules (CAMs) by flow cytometry and immunohistochemistry, and transcription factors, by western blotting.

**Results:**

20 nm cit-AuNP treatment reduced leukocyte and platelet adhesion to cerebral blood vessels, prevented BBB failure, reduced TNF- concentration in brain, and ICAM-1 expression both in circulating polymorphonuclear (PMN) leukocytes and cerebral blood vessels of mice with sepsis. Furthermore, 20 nm cit-AuNP did not interfere with the antibiotic effect on the survival rate of mice with sepsis.

**Conclusions:**

Cit-AuNP showed important anti-inflammatory properties in the brain of mice with sepsis, being a potential candidate to be used as adjuvant drug along with antibiotics in the treatment of sepsis to avoid SAE 
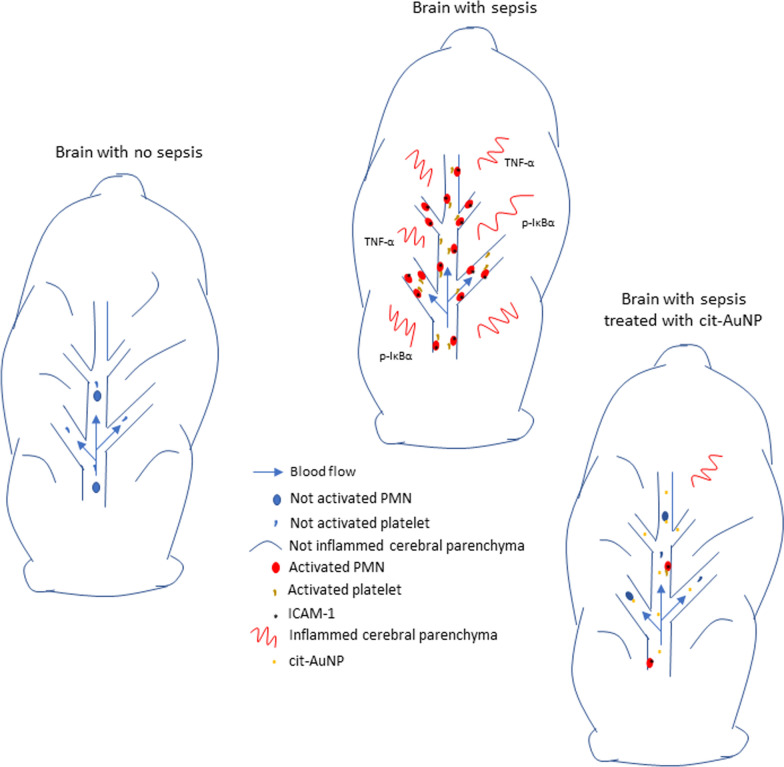

## Background

Sepsis is an emergency medical condition that can lead to death and it is defined as a life-threatening organ dysfunction caused by immune dysregulation in response to an infection [1]. Epidemiological studies show that despite of all clinical and therapeutic efforts, mortality caused by sepsis is still high, and this number is even higher in poor countries [2–4]. Several organs are dramatically affected by sepsis, such as liver, kidneys, lung, gut, and brain [5].

Brain dysfunction during sepsis is widely known as sepsis associated-encephalopathy (SAE) and is mostly caused by a sepsis-induced systemic inflammatory response. Epidemiologically, studies report SAE in 14–63% of septic patients and suggest the large variability observed is due to differences in sepsis severity, SAE diagnostic criteria and individual patient characteristics [6, 7]. Main SAE symptoms are not specific and usually include acute impairment of consciousness, delirium and/or coma, along with electroencephalogram (EEG) changes [8, 9]. Other signs may include hallucinations, disturbances of sleep-wake cycle and agitation. Then, SAE diagnosis is manly performed by exclusion in all patients with delirium of unknown origin [9]. Poor prognosis along with increasing economic burden are frequently associated with SAE [10–13]. For those who recover from sepsis and SAE, impaired cognitive function, mobility and quality of life are often observed months to years after hospital discharge [14, 15], and there is no treatment available today to prevent that.

Inflammation and oxidative stress are key players for the SAE pathophysiology [16]. Two main sites contribute to sense systemic inflammation in brain when the blood brain barrier (BBB) is still intact: the circumventricular organs (CVO) and the vagus nerve [17–19]. Several receptors involved in the immune system function have been reported in the CVO of animals, for instance: interleukin 1 receptor (IL-1R), IL-6R, tumor necrosis factor alpha (TNFα) receptor, Toll-like receptor 4 (TLR4) and cluster of differentiation 14 (CD14) [20, 21]. TLR4 and CD14 are receptors that recognize the lipopolysaccharide (LPS) endotoxin, a component of the cell wall of Gram-negative bacteria [17, 22]. LPS leads to synthesis and release of pro-inflammatory mediators such as TNFα, IL-1β, IL-6, and nitric oxide (NO) [22, 23]. These mediators play important roles in endotoxin-induced responses in brain such as fever, anorexia, sleep, behavioral and neuroendocrine changes [19, 24]. Along with the CVO, the vagus nerve senses peripheral inflammation through its axonal cytokine receptors. The signal is then transmitted to the central nervous system (CNS), particularly to the nucleus of the solitary tract, which controls the baroreflex and is connected to autonomic structures and hypothalamus [25]. Vagotomy prevents fever induced by endotoxin, TNF or IL-1. Progression of sepsis results in BBB failure and further cerebral inflammatory injury [26]. Contribute to BBB breakdown IL-1, TNF and NO [27]. Microglial cells and astrocytes are then activated and release several mediators to keep the inflammation ongoing [28]. NO probably contributes to neurons toxicity as well by triggering their apoptosis [29, 30]. NO-mediated neuronal death seems to be mediated by mitochondrial respiratory chain blockade, and oxidative stress, as consequence of the peroxinitrite—reactive oxygen species (ROS)—formation [31]. Thus, inflammation and oxidative stress are fundamental for the septic encephalopathy full phenotype.

Gold nanoparticles have been progressively demonstrated to own important anti-inflammatory properties. We demonstrated that 20 nm citrate-conjugated gold nanoparticles (cit-AuNP) prevented leukocyte adhesion to vascular endothelial cells after surgery-induced leukocyte chemotaxis in mesentery of Wistar rats [32]. The same way, it was demonstrated that 15 to 20 nm cit-AuNP reduced the number of leukocytes in LPS-stimulated peritoneum or in the collagen type II immunization-induced knee joint lesion in Wistar rats [33–35]. Anti-inflammatory action of 15 to 20 nm cit-AuNP seems to involve reduction in inflammatory cytokines concentration, increase in anti-inflammatory cytokines concentration, reduction in cyclooxygenase type 2, in great part by modulating the expression/activity of transcription factors, mainly the nuclear transcription factor kappa B (NF-κB) [33–38]. It was also reported 20 nm cit-AuNP reduce oxidative stress, in part by reducing the inducible nitric oxygen synthase (iNOS) expression, and increasing glutathione and antioxidant enzymes activity [34, 35, 37–40]. Together, the above-mentioned pharmacological features of the 20 nm cit-AuNP have largely contributed to their success in treating several different animal models of disease in which inflammation and oxidative stress play important roles, such as, colitis, Alzheimer’s disease, and rheumatoid arthritis [33, 34, 38, 40]. Concomitantly injected in mice with antibiotic treatment, 21 nm cit-AuNP showed both reduced mortality and systemic inflammatory parameters in mice with sepsis [41]. However, nothing is known regarding the effect of 20 nm cit-AuNP treatment in the sepsis induced-inflammation in brain of mice. Once the BBB is compromised, we aim to test whether 20 nm cit-AuNP can alleviate the acute changes caused by sepsis in brain of mice, with focus on inflammation.

## Results

### Gold nanoparticles

Concentration of the colloidal cit-AuNP solution provided was 6.5 × 10^11^ nanoparticles/mL in Milli-Q H_2_O, the average size was 20.1 ± 2.4 nm, and zeta potential of –26.1 mV. This zeta potential indicates high stability in solution.

### Leukocyte and platelet adhesion in pial microvessels

**Fig. 1:**
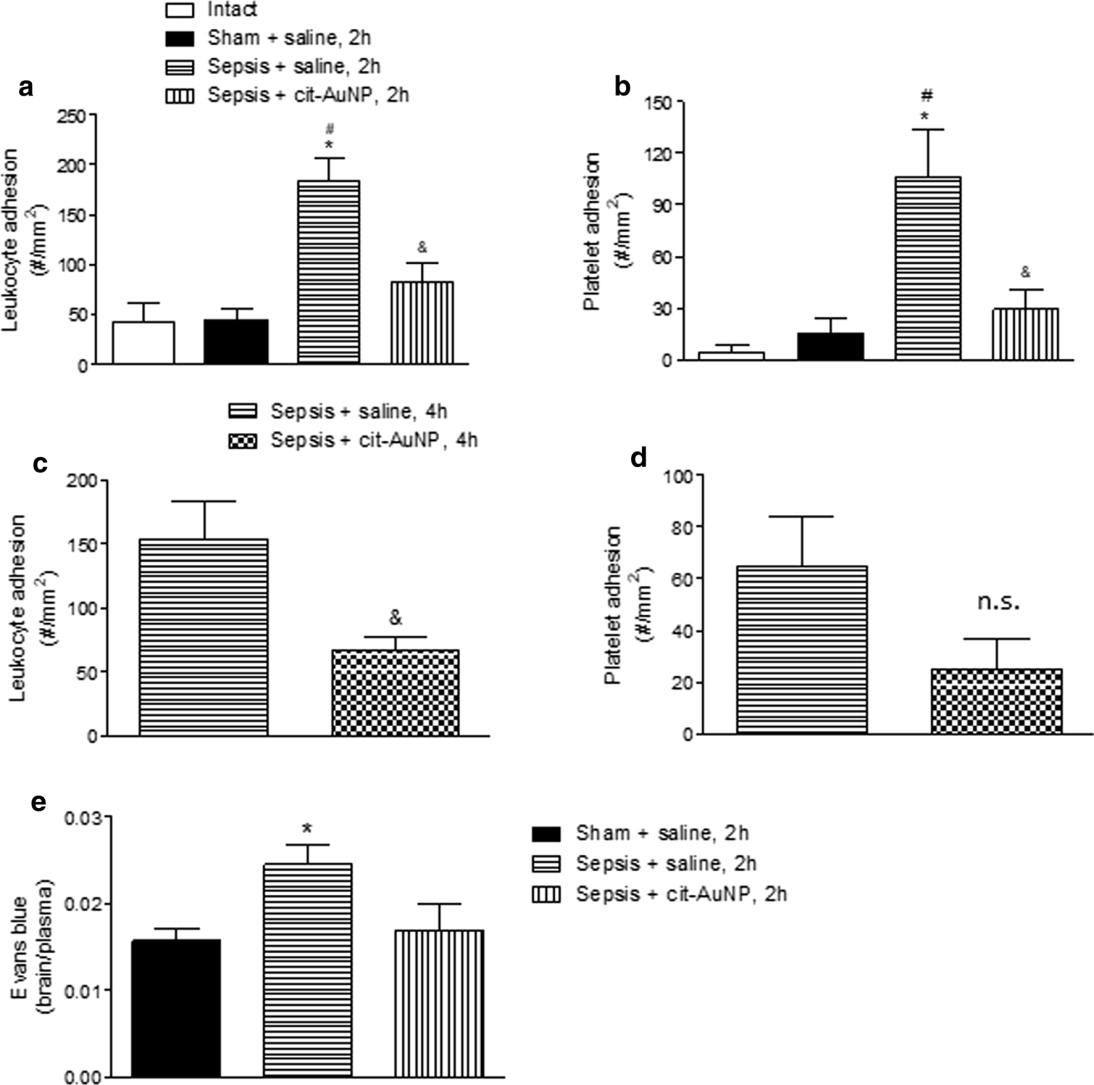
Effect of citrate-covered gold nanoparticles (cit-AuNP) on leukocyte and platelet adhesion and blood brain barrier. Twenty nanometers cit-AuNP or saline was injected intravenously (IV) 2 (**a**, **b**, and **e**) or 4 h (**c** and **d**) after induction of sepsis or the sham-operated procedure in mice. Intact group was submitted to no surgery or treatment. Leukocyte (**a** and **c**) and platelet (**b** and **d**) adhesion to pial microvessels and BBB permeability assay (**e**) were performed 6 h after induction of sepsis. Sepsis led to enhanced leukocyte and platelet adhesion to pial vessels, and cit-AuNP treatment reduced those parameters. Sepsis also led to BBB failure, but that BBB compromising was not observed in mice previously treated with cit-AuNP. * *P* < 0.05 vs. Intact; ^#^ *P* < 0.05 vs. Sham + saline; ^&^ *P* < 0.05 vs. sepsis + saline. ANOVA followed by Tukey’s test was used for comparison when more than two groups were assayed, and unpaired t test was used for comparison between two groups. Five to ten animals per group were used. n.s., not significant

Increase in leukocyte and platelet adhesion in pial microvessels was observed 6 h after sepsis induction in mice compared to both the intact and sham groups (Fig. 1a and b). Mice treated with cit-AuNP 2 h after sepsis induction did not show enhanced leukocyte and platelet adhesion in pial microvessels (Fig. 1a and b). The same result was observed in mice treated with cit-AuNP 4 h after sepsis induction (Fig. 1c and b). Once we did not observe differences in leukocyte and platelet adhesion in cerebral microvessels of mice from the intact and sham groups, next experiments had the sham-operated mice as the only control group.

### Evans blue extravasation in brain

Sepsis enhanced the EB extravasation in brain of mice observed 6 h after induction compared to the sham group (Fig. 1e). Cit-AuNP injected 2 h after induction of sepsis avoided increase in Evans blue blood vessels permeation (Fig. 1e).

### Cytokines concentration in brain

**Fig. 2:**
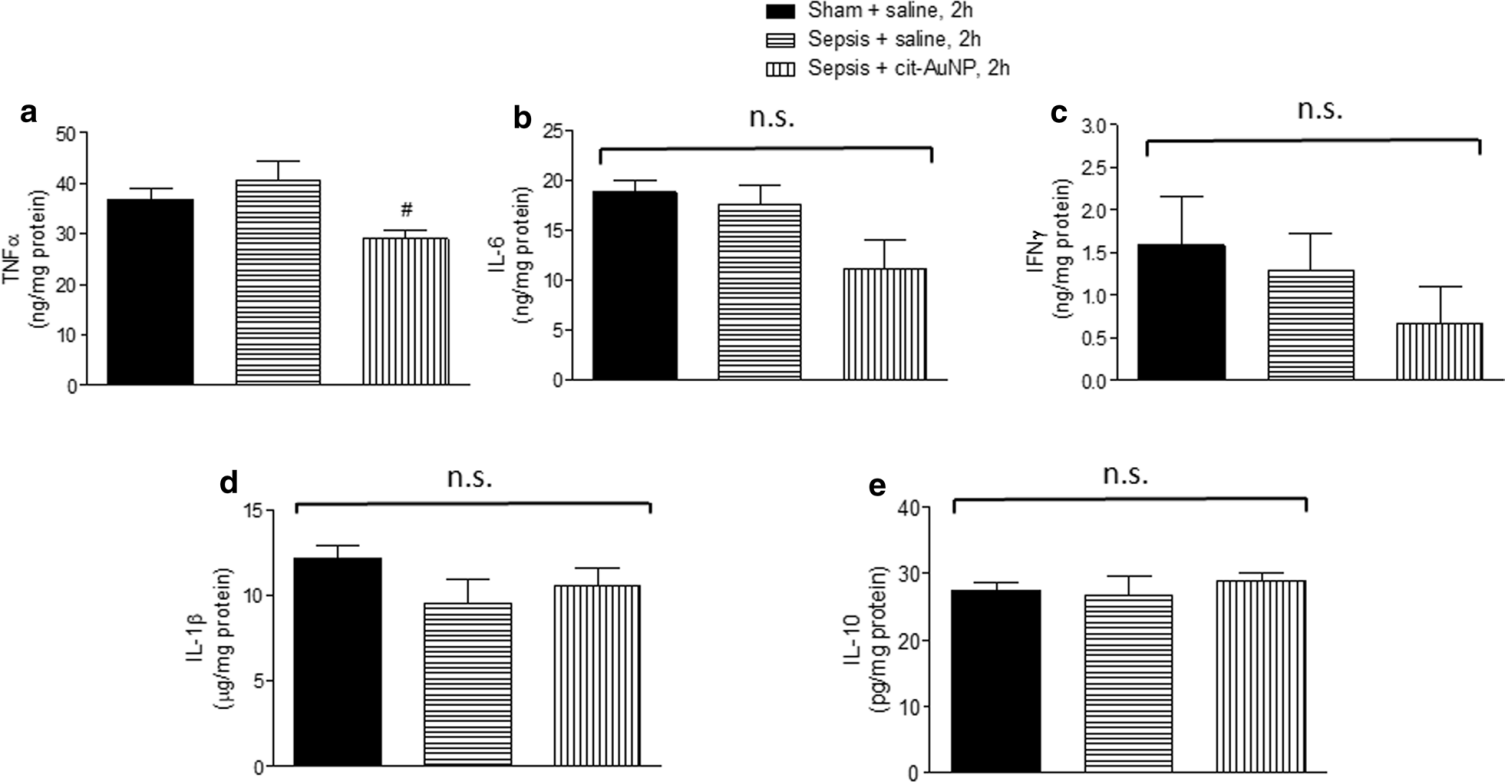
Effect of citrate-covered gold nanoparticles (cit-AuNP) on cerebral cytokines in mice with sepsis. Twenty nanometers cit-AuNP or saline was injected intravenously (IV) 2 h after induction of sepsis or the sham-operated procedure in mice. No significant change in tumor necrosis factor alpha (TNFα) (**a**), interleukin 6 (IL-6) (**b**), interferon gamma (IFNγ) (**c**), IL-1β (**d**), or IL-10 (**e**) was noticed in the cerebral tissue 6 h after induction of sepsis. Cit-AuNP treatment reduced TNFα concentration, but not other cytokines. ^#^ *P* < 0.05 vs. Sepsis + saline. ANOVA followed by Tukey’s test was used for comparison among groups. Five to six animals per group were used. n.s., not significant

TNFα, IL-6, IFNγ, IL-1β and IL-10 were measured in brain of mice 6 h after induction of sepsis. We observed none of those cytokines were changed in brain of mice with sepsis compared to the sham group at that time (Fig. 2). Cit-AuNP treatment performed 2 h after induction of sepsis reduced cerebral TNFα concentration, but not other cytokines, compared to mice with sepsis treated with saline (Fig. 2).

**Fig. 3:**
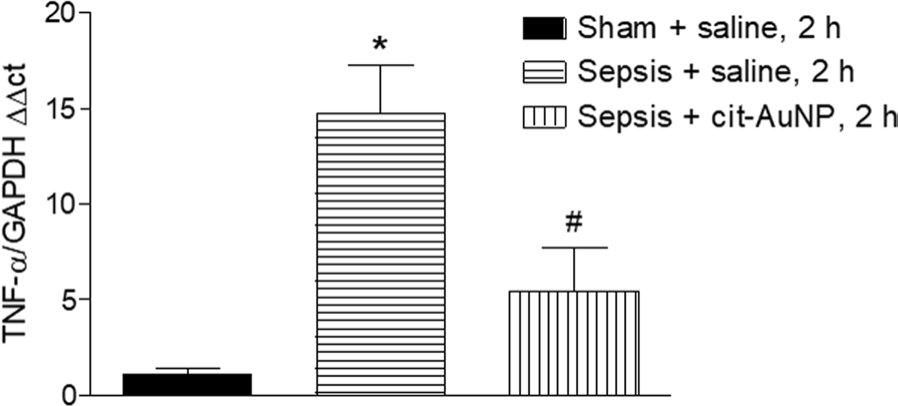
Effect of citrate-covered gold nanoparticles (cit-AuNP) on TNFα mRNA in brain of mice with sepsis. Twenty nanometers cit-AuNP or saline was injected intravenously (IV) 2 h after induction of sepsis or the sham-operated procedure in mice. Enhanced TNFα mRNA expression was observed in the cerebral tissue 6 h after induction of sepsis, and cit-AuNP treatment reduced it. * *P* < 0.05 vs. sham; ^#^ *P* < 0.05 vs. Sepsis + saline. ANOVA followed by Tukey’s test was used for comparison among groups. Five to six animals per group were used

By doing real time PCR, we observed sepsis caused increase in TNFα mRNA in brain 6 h after induction, and cit-AuNP treatment performed 2 h after induction of sepsis reduced TNF mRNA levels in the cerebral tissue (Fig. 3).

### Cell adhesion molecules expression

**Fig. 4:**
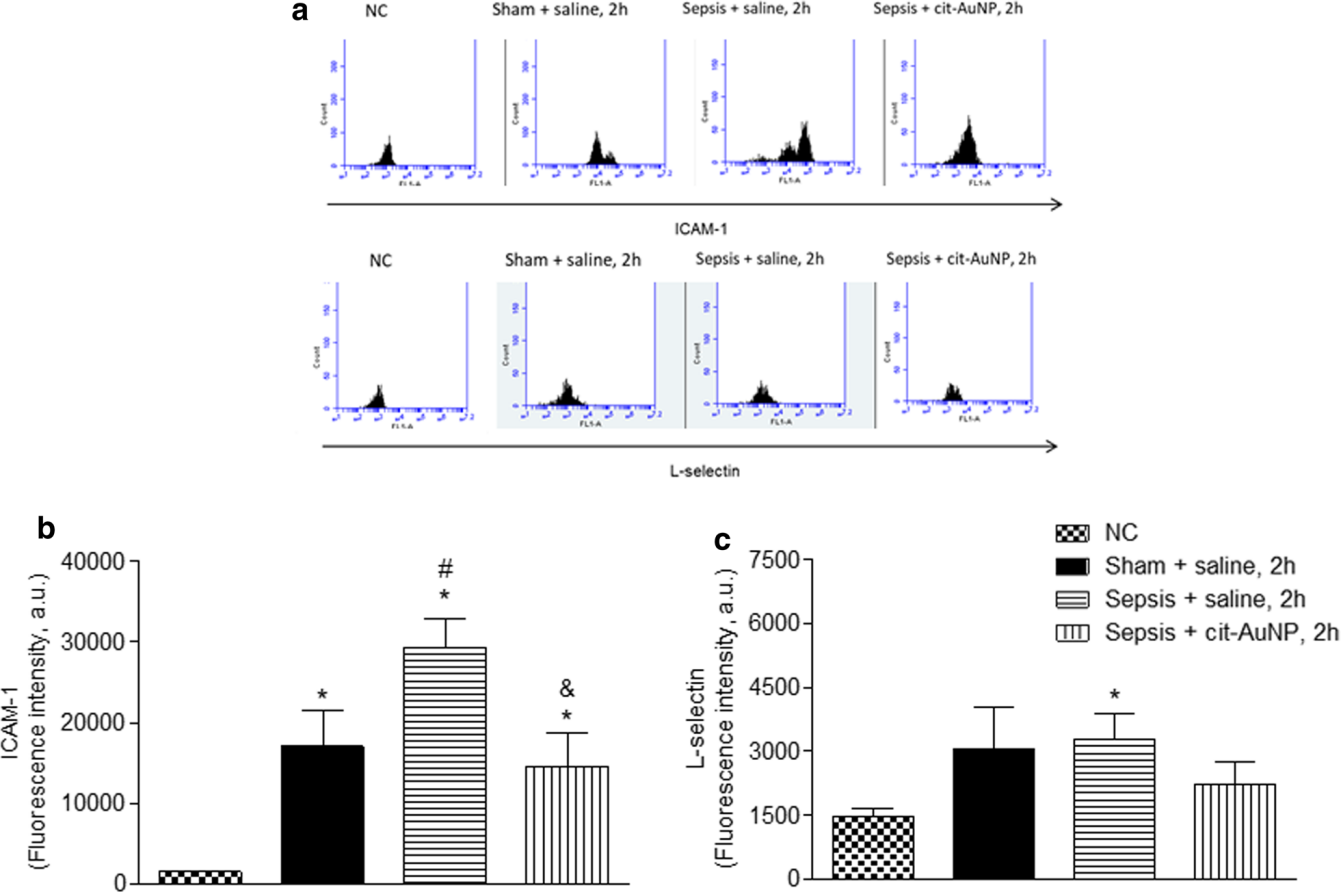
Effect of citrate-covered gold nanoparticles (cit-AuNP) on ICAM-1 and L-selectin in circulating polymorphonuclear (PMN) leukocytes. Twenty nanometers cit-AuNP or saline was injected intravenously (IV) 2 h after induction of sepsis or the sham-operated procedure in mice. **a** Representative flow cytometry graphs of ICAM-1 (upper part) and L-selectin (lower panel) expressions in PMN leukocytes of mice with sepsis or sham-operated previously treated with 20 nm cit-AuNP or saline. **b** Quantification of the ICAM-1 expression in PMN leukocytes of mice with sepsis or sham-operated previously treated with 20 nm cit-AuNP or saline. **c** Quantification of the L-selectin expression in PMN leukocytes of mice with sepsis or sham-operated previously treated with 20 nm cit-AuNP or saline. Enhanced ICAM-1, but not L-selectin, expression was quantified in PMN leukocytes 6 h after induction of sepsis, and cit-AuNP treatment reduced it. * *P* < 0.05 vs. negative control (NC); ^#^ *P* < 0.05 vs. Sham + saline; ^&^ *P* < 0.05 vs. Sepsis + saline. ANOVA followed by Tukey’s test was used for comparison among groups. Seven to ten animals per group were used. a. u., arbitrary units

ICAM-1 and L-selectin were measured in circulating polymorphonuclear (PMN) leukocytes 6 h after induction of sepsis in mice. Sepsis increased ICAM-1, but not L-selectin, expression in circulating PMN leukocytes compared to the sham group (Fig. 4a and b). Cit-AuNP treatment performed 2 h after induction of sepsis reduced the ICAM-1 and did not change de L-selectin expression in PMN leukocytes compared to saline-treated mice (Fig. 4a and b).

**Fig. 5:**
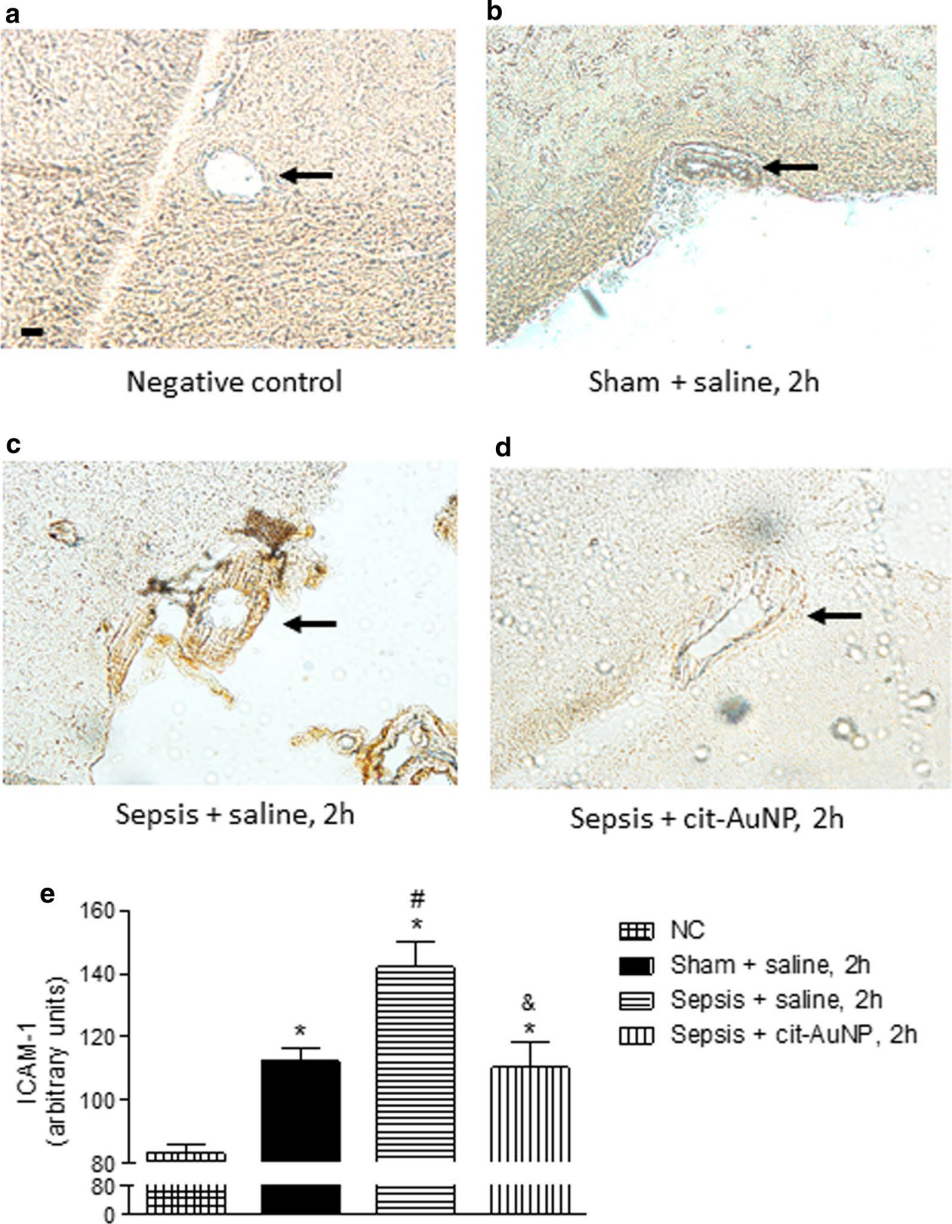
Effect of citrate-covered gold nanoparticles (cit-AuNP) on ICAM-1 in cerebral vessels of mice with sepsis. Twenty nanometers cit-AuNP or saline was injected intravenously (IV) 2 h after induction of sepsis or the sham-operated procedure in mice. Representative photos of the ICAM-1 expression in cerebral blood vessels (black arrows) from a negative control (NC) (in which no anti-ICAM-1 antibody was added in the assay) (**a**), sham-operated mice treated with saline (**b**), mice with sepsis treated with saline (**c**), and mice with sepsis treated with cit-AuNP (**d**). **e** Quantification of the ICAM-1 expression in the cerebral blood vessels. Enhanced ICAM-1 expression in cerebral blood vessels was noticed 6 h after induction of sepsis, and cit-AuNP treatment reduced it. * *P* < 0.05 vs. NC; ^#^ *P* < 0.05 vs. Sham + saline; ^&^ *P* < 0.05 vs. Sepsis + saline. ANOVA followed by Tukey’s test was used for comparison among groups. Five to six animals per group were used. Bar, 10 µm

ICAM-1 expression was measured in cerebral microvessels of mice 6 h after sepsis induction as well. Enhanced ICAM-1 expression was observed in cerebral microvessels of mice with sepsis compared to sham-operated mice (Fig. 5). Cit-AuNP treatment performed 2 h after induction of sepsis reduced ICAM-1 expression in cerebral microvessels of mice compared to saline-treated mice (Fig. 5).

### Transcription factors and transcription factors related‐proteins expression in brain

**Fig. 6:**
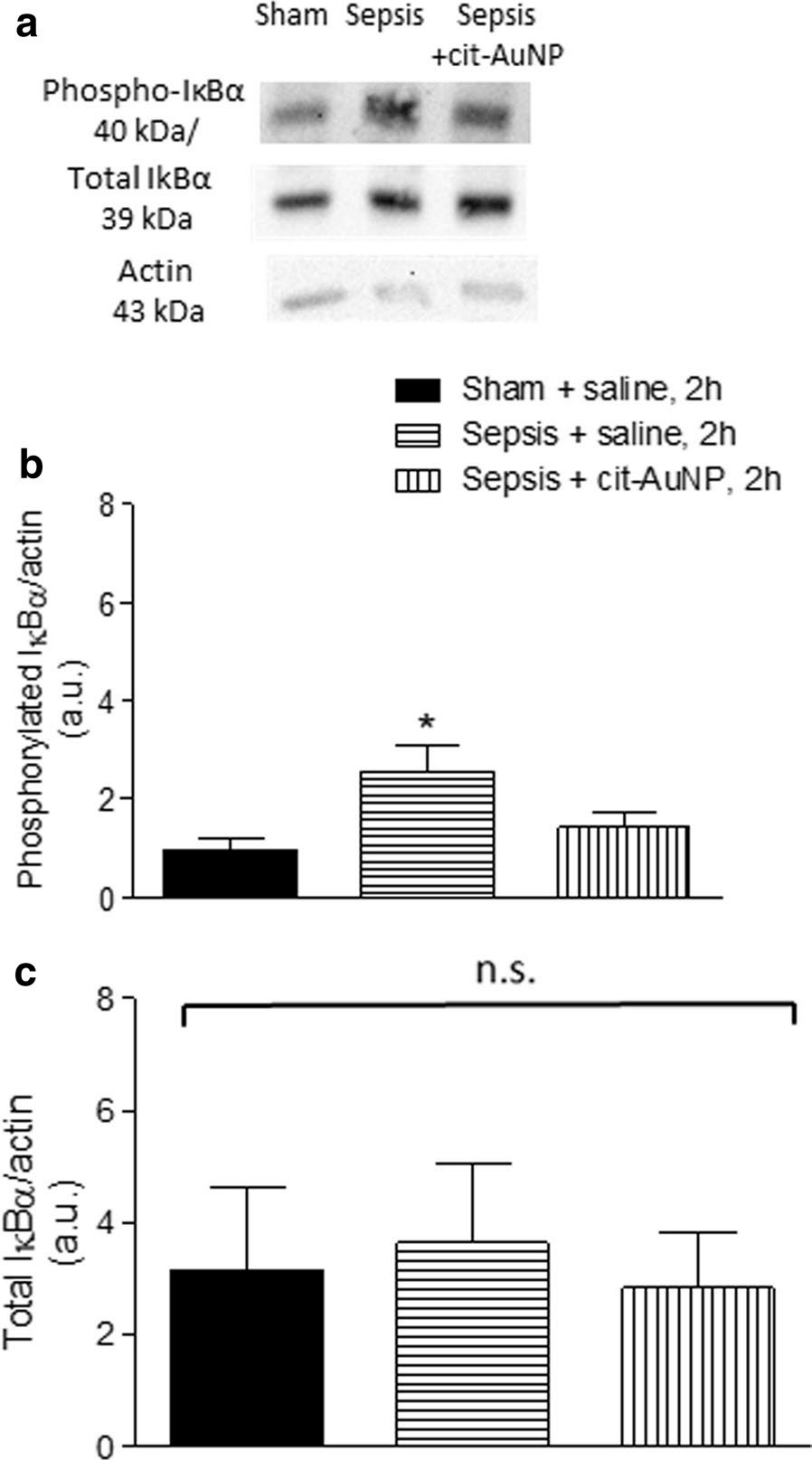
Effect of citrate-covered gold nanoparticles (cit-AuNP) on phosphorylated and total inhibitor κBα (IκBα) in brain. Twenty nanometers cit-AuNP or saline was injected intravenously (IV) 2 h after induction of sepsis or the sham-operated procedure in mice. Representative blots (upper panel) and quantification of the phosphorylated IκBα (phospho-IκBα) (middle panel) and total IκBα (lower panel) expressions in brain of mice with sepsis or sham-operated previously treated with saline or cit-AuNP. Enhanced phospho-IκBα, but not total IκBα, was demonstrated in brain of mice 6 h after induction of sepsis, but that increase was not observed in mice previously treated with cit-AuNP. * *P* < 0.05 vs. Sham + saline. ANOVA followed by Tukey’s test was used for comparison among groups. Four to five animals per group were used. n. s., not significant; a. u., arbitrary units

Transcription factors and related proteins were measured in brain of mice 6 h after induction of sepsis. We observed enhanced expression of phosphorylated IκBα, but not total IκBα, in brain of mice with sepsis compared to the sham-operated mice (Fig. 6). Mice with sepsis previously treated with cit-AuNP did not show enhanced phosphorylated IκBα expression in brain compared to the sham group (Fig. 6). Cit-AuNP treatment did not change the total IκBα expression in brain of mice with sepsis (Fig. 6).

Regarding to the transcription factors AP1 and HIF-1α, sepsis did not change their expression in brain of mice [see Additional file 1]. Cit-AuNP treatment performed 2 h after sepsis induction did not change the AP1 and HIF-1α expressions in brain of mice as well [see Additional file 1].

### Survival curve

**Fig. 7:**
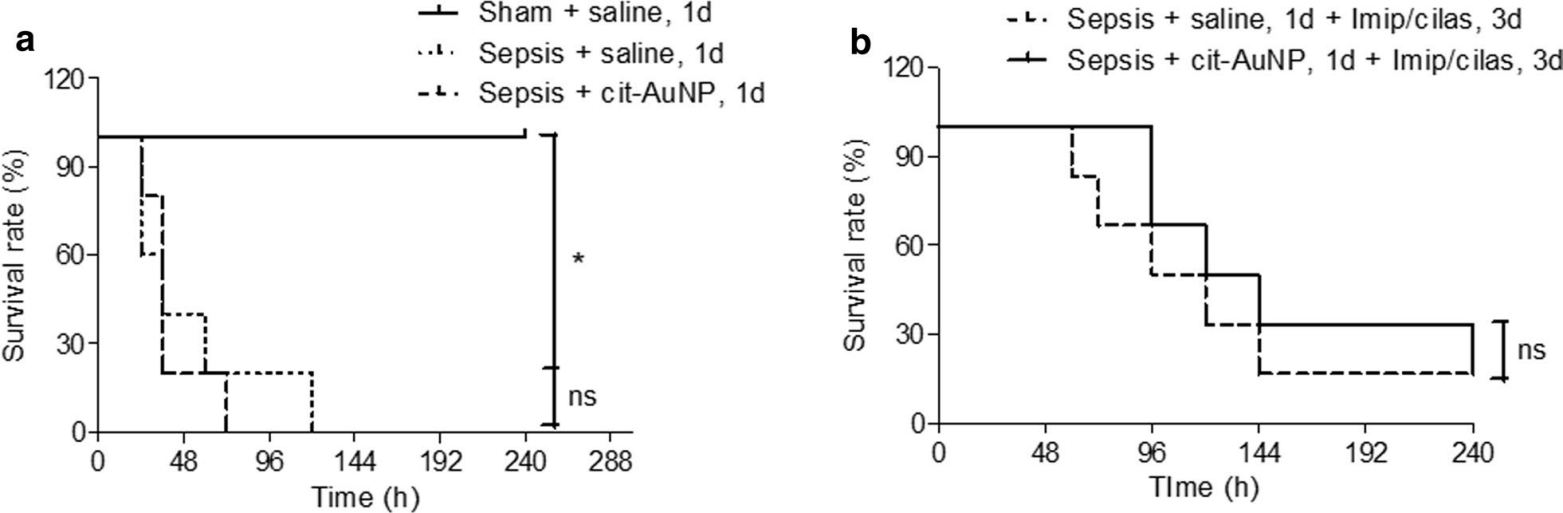
Effect of citrate-covered gold nanoparticles (cit-AuNP) and imipenem and cilastatin on the survival rate. Twenty nanometers cit-AuNP or saline was injected intravenously (IV) 2 h and 24 h (named one day − 1d −) after induction of sepsis or the sham-operated procedure in mice, and 0.3 mg imipenem and 0.6 mg cilastatin, per animal, were injected twice a day for three days (3d), starting 2 h after induction of sepsis and at 12 h intervals afterwards. Cit-AuNP alone (**a**) or combined with imipenem and cilastatin (**b**) did not change the survival rate of mice with sepsis. * *P* < 0.05 vs. Sham + saline. Log-rank (Mantel-Cox) test was used for comparison of survival curves. Five to six animals per group were used. n. s., not significant

Mice with or without sepsis (sham group) were treated with saline or cit-AuNP and followed for ten days in order to measure the survival rate. We observed that all mice with sepsis did not survive longer than five days and cit-AuNP did not change the survival rate compared to saline-treated mice (Fig. 7a). Conversely, all sham-operated mice survived during the whole observation time (Fig. 7a).

When cit-AuNP or saline was combined with the antibiotic treatment regimen (imipenem/cilastatin), 17% of survival was observed in mice with sepsis after 10 days of observation in both groups (Fig. 7b). Thus, no significant difference was observed when cit-AuNP was combined with antibiotics compared to saline and antibiotics treatment regimen.

## Discussion

In this study we observed 20 nm cit-AuNP treatment reduced leukocyte and platelet adhesion in pial vessels of mice with sepsis, alleviated BBB failure, and, in parallel, reduced TNFα concentration and phosphorylated IκB expression in the brain parenchyma, ICAM-1 expression in both PMN leukocytes and cerebral blood vessels, and did not interfere with the effect of imipenem/cilastatin on the survival rate of mice with sepsis.

In an elegant in vitro experiment, it was demonstrated that an experimental cytokine mixture, which mimicked plasma obtained from patients with severe sepsis, led to enhanced human leukocytes adhesion to human cerebrovascular endothelial cells (hCMEC/D3) [42]. Similarly, increased leukocyte and platelet adhesion to cerebral vessels is a common finding in animal models of SAE [43–47]. Corroborating those results, we observed sepsis increased both leukocyte and platelet adhesion to pial vessels of mice. Regarding to the effect of 20 nm cit-AuNP on the leukocyte adhesion, we demonstrated in a previous study enhanced leukocyte adhesion in mesentery microvessels of rats submitted to laparotomy, and previous treatment with 20 nm cit-AuNP prevented that leukocyte-endothelial cells interaction [32]. Now, we report that 20 nm cit-AuNP reduces not only leukocyte adhesion but also platelet adhesion in pial vessels of mice 6 h after induction of sepsis. Of importance, reduction of leukocyte and platelet adhesion were observed in two different treatment protocols: cit-AuNP injected either 2 h or 4 h after induction of sepsis. This implies in a larger therapeutic window. Leukocyte adhesion is commonly accompanied by platelet adhesion during an inflammatory insult. CAMs are greatly responsible for that interaction [48]. Along with reduction in leukocyte adhesion, it is usual to occur lower platelet adhesion once they interact each other. Several studies show parallel reduction in both leukocyte and platelet adhesion to microvessels after a therapeutic intervention [49–51]. Apart from the study we published few years ago [32], no reports have investigated the effect of gold nanoparticles on the leukocyte adhesion so far and even less on the platelet adhesion under any circumstance. Despite of that, it has long been known the anti-adhesive leukocyte-endothelial cell property of molecular gold. It was showed gold sodium thiomalate and auranofin impaired interleukin 1 beta (IL-1β)-induced human umbilical vein endothelial cells (HUVEC) to bind PMN cells by diminishing E-selectin and/or ICAM-1 expression, in vitro [52]. Not only PMN leukocyte adhesion, but also PMN leukocyte migration is inhibited by gold ions, as demonstrated in [53]. This anti-adhesive property of molecular gold reducing the leukocyte-endothelial cell interaction helps to understand now the past clinical use of gold compounds in the treatment of inflammatory disease, mainly rheumatoid arthritis [54, 55]. Reduction of migration induced by gold nanoparticles is also observed in several other cell types besides PMN leukocytes, like endothelial cells or cancer cells [56–58], what makes gold nanoparticles potential treatment not only for inflammatory conditions but also other diseases, as cancer.

BBB damage in patients with sepsis was observed using different strategies: (i) post-mortem [59]; (ii) after incubating hCMEC/D3 cells, as a model of BBB, in presence of serum from septic patients [60]; and (iii) indirectly, by measuring plasma S100B, a marker of BBB lesion [61]. BBB failure is often reported in animal models of sepsis as well [62–64]. Corroborating those reports, we observed enhanced BBB permeability in mice 6 h after induction of sepsis. Furthermore, we demonstrated besides reducing leukocyte and platelet adhesion to pial vessels, 20 nm cit-AuNP treatment prevented that BBB failure. This is the first study to show the anti-edema effect of 20 nm cit-AuNP. Prolonged leukocytes and endothelial cells interaction can greatly increase blood vessels permeability [65]. Then, reducing leukocyte adhesion to pial vessels may have accounted for the diminished BBB failure we observed after cit-AuNP treatment. Effect of 20 nm cit-AuNP reducing vascular leakage may be important in other conditions where edema greatly contributes to disease phenotype, like in ischemic stroke and cerebral trauma. Additional studies can address that hypothesis.

According to our results, the therapeutic effect of 20 nm cit-AuNP reducing leukocyte and platelet adhesion to pial vessels and edema in brain of mice 6 h after induction of sepsis may come at least in part from its action reducing TNFα concentration in brain and, in a larger extension, by diminishing the ICAM-1 expression both in leukocytes and cerebral blood vessels. Studies show TNFα concentration in brain either increases or does not change after induction of sepsis in mice. It was reported moderate increase in TNFα concentration in brain of mice 6 h after induction of sepsis and that increase was higher 12 h up to 7 days after induction [66]. Other reports showed increase in TNFα concentration 3 h and 4 h, respectively, but not 24 h after LPS injection in mice [67, 68]. On the other hand, another study showed no increase in TNFα concentration 0.5, 1, 2, 4, and 24 h in brain of mice after LPS injection [69]. Here we demonstrated a great increase in TNFα mRNA expression and a slight and not significant increase in TNFα protein concentration in brain of mice 6 h after induction of sepsis, and 20 nm cit-AuNP treatment reduced both TNFα protein and mRNA levels. This is the first time it is reported decrease in TNFα levels in brain of mice after 20 nm cit-AuNP treatment, although the effect of 20 nm cit-AuNP reducing TNFα concentration in other tissues was previously demonstrated by others. In fact, it was observed reduced TNFα circulatory concentration after concomitant 21 nm cit-AuNP and antibiotic injection 18 h after induction of sepsis in mice compared to mice with sepsis that received only antibiotic treatment [41]. Twenty nanometers cit-AuNP intraperitoneally injected for 20 days reduced TNFα concentration in serum of arthritic rats [34]. Thirty nanometers cit-AuNP topically applied reduced the enhanced TNFα concentration in aqueous humor of rats 24 h after LPS-induced uveitis compared to saline-treated rats [70]. Not only protein concentration, but also TNF mRNA are reduced after cit-AuNP treatment in liver and fatty tissue of mice fed high fat diet (HFD) for 28 days and treated for nine weeks with 21 nm cit-AuNP compared to HFD mice that received only vehicle [71].

CAMs are of fundamental importance to leukocyte and endothelial cell interaction and participate in all steps of the diapedesis process, i.e., rolling, crawling, firm adhesion, and migration. Of those, ICAM-1 plays a critical role for the leukocyte adhesion to endothelial cells in several tissues, including in cerebral blood vessels of mice after induction of sepsis [72], or in an in vitro model of interaction of human PMN and human cerebrovascular endothelial cells, in which PMN were previously stimulated with plasma from severe sepsis patients [42]. Similarly, we observed enhanced ICAM-1 expression in cerebral blood vessels of mice 6 h after induction of sepsis. Additionally, increase in ICAM-1 expression in PMN was also demonstrated. Enhanced ICAM-1 expression in pial vessels induced by sepsis may explain the increase in leukocyte adhesion in our model. Different from the adhesive role of ICAM-1 in blood vessels, enhanced ICAM-1 expression on PMN is a hallmark of leukocyte activation—mainly enhanced phagocytosis and ROS production—as demonstrated in [73]. Additionally, an important role of ICAM-1^+^ neutrophils was recently reported in sepsis. It was demonstrated ICAM-1 activation in neutrophils triggers neutrophil extracellular traps (NET) formation and then results in exaggerated sepsis-induced inflammation [74]. Then, increase in ICAM-1 expression in PMN leukocytes we demonstrated 6 h after induction of sepsis probably contributed for the disease progression. Our results also showed 20 nm cit-AuNP reduced ICAM-1 expression both in leukocytes and cerebral blood vessels. In this context, it was demonstrated gold nanoparticles reduce TNF-α-induced CAM in both human umbilical vein endothelial cells and aortic endothelial cells by increasing their ubiquitination, i.e., degradation [75]. The same seems to happen in PMN leukocytes, but probably not in cerebral endothelial cells, once we observed reduction in ICAM-1 expression in circulating PMN 4 h after intravenous treatment with cit-AuNP but not appreciable amount of cit-AuNP in the cerebral tissue of cit-AuNP-treated mice [see Additional file 1]. Cit-AuNP would be expected to be seen in the cerebral tissue to explain a direct action of cit-AuNP enhancing the ICAM-1 degradation in endothelial cells. Thus, 20 nm cit-AuNP probably acted indirectly on cerebral endothelial cells reducing their inflammatory profile after reducing activation of circulatory PMN leukocytes. In fact, activation of endothelial cells by circulating leukocytes have been reported for a long time [76–78]. Additionally, we observed increase in phosphorylated IκBα expression in brain of mice 6 h after induction of sepsis, and 20 nm cit-AuNP treatment reduced the enhanced expression of that phosphorylated transcription modulator. IκBα phosphorylation allows NF-κB migrates to the nucleus and bind to the DNA to transcribe pro-inflammatory molecules [79]. Then, cit-AuNP probably also indirectly mediated reduction in phosphorylated IκBα, leading to lower NF-κB migration to the nucleus and lowering expression of molecules that participate in the inflammatory process, such as ICAM-1 and TNFα. As we commented above, we observed both lower TNFα concentration in brain and expression of ICAM-1 in circulating PMN and pial vessels of mice with sepsis previously treated with cit-AuNP.

Regarding to the effect of 20 nm cit- AuNP on the survival rate of animal with sepsis, we did not observe change of it, i.e., no mice survived after five days of sepsis induction both after cit-AuNP or saline treatment. The same way, when 20 nm cit-AuNP and antibiotic (imipenem/cilastatin) were injected in combination, gold nanoparticles did not interfere with the survival rate after 10 days of observation, i.e., no significant difference was observed compared to the group treated only with the antibiotic regimen. No interference in the antibiotic effect on the survival rate is an advantage of 20 nm cit-AuNP concomitant treatment once they would probably be used together in a sepsis patient. On the other hand, slight improvement in the survival rate was observed when 21 nm cit-AuNP was combined with imipenem/cilastatin treatment, however, the observation period they used was 4 days while we observed mice 10 days after induction of sepsis [41]. Thus, there is a possibility that after ten days of observation, that difference reported could not be seen anymore.

## Conclusions

In the present study, we show for the first time the beneficial effect of 20 nm cit-AuNP in reducing brain inflammation induced by sepsis. Besides, 20 nm cit-AuNP did not interfere with the antibiotic effect on the survival rate of mice with sepsis, what makes 20 nm cit-AuNP a potential candidate to be used as adjuvant along with antibiotics in the treatment of sepsis, in order to avoid SAE.

## Methods

### Gold nanoparticles

Gold nanoparticles used in this study were ordered from the Nitparticles Company (catalog number #510,006,160, Zaragoza, Spain). Cit-AuNP were diluted in Milli-Q H_2_O. Citrate was used as a reducing agent to form and stabilize the gold nanoparticles in solution, avoiding aggregates formation and, then, changes in nanoparticles diameter, as described in [80]. Cit-AuNP stability is one year in fridge, according to the manufacturer.

### Animals

Seven-week-old specific-pathogen-free (SPF) female C57BL/6 mice (18–22 g body weight) were ordered from the Medical School Animal Facility Network at the University of Sao Paulo (USP). They were housed in the vivarium of the Department of Pharmacology in the Institute of Biomedical Sciences (IBS) until use. Mice were housed in a 12 h/12 h light-dark cycle, controlled room temperature (22 to 24  ^o^C) and humidity (30 to 70%). They were fed chow and water *ad libitum* until use. All efforts were made to minimize suffering and to reduce the number of animals used.

### Sepsis induced by cecal ligation and puncture

Sepsis was induced in eight- to ten-week-old mice by doing the cecal ligation and puncture (CLP) method, according to [81]. In brief, mice were anesthetized with inhaled 2% isoflurane (Isoforine®, Cristalia, Itapira, SP, Brazil), abdominal upper midline incision was performed, and cecum exteriorized. Cecum was ligated 1 mm distal from the ileocecal valve using a 6−0 nylon thread and punctured twice (middle and distally) using a 21G needle. Few feces extravasated. Cecum was gently put back the abdominal cavity and muscle and skin individually closed using a 6−0 nylon suture. Mice were rehydrated by subcutaneously injecting 1 mL saline. Aseptic material was used for the procedure that lasted about 15 min. Mice were observed until recovering from anesthesia and experiments were performed 6 h after induction of sepsis. Similar protocol was done in sham animals, but no ligation and puncture were carried out. One intact group was added in which no surgery was done.

### Gold nanoparticle treatment

Two hours after induction of sepsis, mice were anesthetized with inhaled 2% isoflurane (Isoforine®) and a small vertical incision performed in the mid inner thigh to intravenously (IV) inject 2.9 × 10^11^ cit-AuNP (150 µL volume). An additional group of mice was treated with cit-AuNP 4 h after induction of sepsis the same dose and site of injection described above. We previously demonstrated that a similar dose of cit-AuNP IV injected reduced the enhanced surgery-induced leukocyte adhesion in mesentery vessels of rats [32]. Similar protocols were used to inject saline in both mice with sepsis and the sham group.

### Mice preparation for pial vessels observation

Leukocytes and platelet adhesion to pial vessels were studied by fluorescence intravital microscopy, as previously described [82]. Briefly, six hours after induction of sepsis or the sham-operated procedure, mice were anesthetized with intraperitoneal (IP) ketamine and xylazine (100 mg/kg and 10 mg/kg body weight, respectively) and a polyethylene microtubing (PE/1, Scientific commodities, Lake Havasu City, AZ, USA) was fixed in the left femoral vein to allowing IV injection of 0.02% 6G-rhodamine (Sigma-Aldrich, St. Louis, MN, USA), labeled platelets and supplemental anesthesia. After craniotomy, exposed brain tissue was immersed in an artificial cerebrospinal fluid and cerebral vessels were observed through the dura mater with an upright fluorescent microscope (DM LFS, Leica, Wetzlar, Germany), using a 40x water immersion lens. Pial venules (20 to 50 µm diameter, 100 µm long) were randomly chosen for observation.

### Platelets isolation and labelling

Approximately 100 × 10^6^ platelets were isolated from a healthy donor mouse, labeled (in green) *ex vivo* with 90 µM carboxyfluorescein diacetate succinimidyl ester (carboxyfluorescein diacetate, succinimidyl ester, mixed isomers, ThermoFisher Scientific, Waltham, MA, USA)—this extracorporeal staining produces minimal activation of platelets—according to the protocol described in [83], and injected in a recipient mice with or without sepsis (sham or intact groups) through the left femoral vein just before observation. Following platelets injection, 100 µL of 0.02% rhodamine 6G was slowly injected (for 5 min) to fluorescently label (in red) circulating leukocytes. Adhesion leukocytes and platelets were defined as cells remaining stationary within venules for 30 seconds. Cell adhesion data are expressed as number of cells per millimeter squared of venular surface, calculated from venular diameter and length, assuming cylindrical geometry.

### Blood brain barrier permeability assay

Permeability of BBB was assessed using the Evans blue (EB) extravasation method, according to the protocol described in [82]. Shortly, 2% EB (Sigma-Aldrich) solution was prepared and injected (4 mL/kg) into the femoral vein just after induction of sepsis or the sham-operated procedure. Two hours later cit-AuNP or saline were injected into the contralateral femoral vein as described above, and assay was carried out 6 h after induction of sepsis. For that, blood (0.4 mL) was collected from the inferior vena cava, and mouse was transcardially perfused with phosphate-buffered saline (PBS) (100 mm Hg) for 5 mins. Brain was removed and separated from the dura mater and cerebellum. Cerebrum was divided into hemispheres, each of which was homogenized and sonicated in 1 mL of 50 % trichloroacetic acid (Sigma-Aldrich), and centrifuged at 12,100x g for 20 mins. The supernatant was diluted with ethanol and the concentrations of EB in brain tissue and plasma were measured using a fluorescence spectrophotometer (FLUOstar Optima microplate reader; BMG LABTECH, Inc, Ortenberg, Germany). The BBB permeability was quantified by dividing tissue EB concentration (µg/g brain weight) by the plasma EB concentration (µg/g).

### Brain and blood collect

Six hours after induction of sepsis or the sham-operated procedure, median longitudinal laparotomy was performed in previously anesthetized mice (100 mg/kg ketamine and 10 mg/kg xylazine I.P. injected), blood collected from the vena cava into a dextrose-citrate solution-containing tube (1:10, vol:vol) (Sigma-Aldrich), which was used to prevent blood clotting, and prepared for measuring cell adhesion molecules (CAMs) expression in leukocytes by flow cytometry. Mice were then transcardially perfused with PBS (100 mmHg) for about 5 mins, brain was harvested, divided into hemispheres, the right one used to measure intercellular adhesion molecule 1 (ICAM-1) by immunohistochemistry, and the left one quickly frozen, and kept in −80^o^C freezer until use, in order to measure cytokines by enzyme-linked immunosorbent assay (ELISA), and phosphorylated or total inhibitor of kappa B alpha (IκBα), activator protein 1 (AP-1), and hypoxia inducible factor 1 alpha (HIF-1α) by western blotting. All those techniques are described ahead.

### Cytokines measurement in brain

Frozen brain was powered and homogenized in lysis buffer (10% RIPA buffer, Merk Millipore, MA, USA), added 1 mM phenylmethylsulfonyl fluoride (PMSF) (Amresco, Solon, OH, USA), 10 mM sodium orthovanadate (Sigma-Aldrich), 100 mM sodium fluoride (LabSynth, Diadema, SP, Brazil), 10 mM sodium pyrophosphate (Sigma-Aldrich), and 0.2% protease inhibitor (P8340, Sigma-Aldrich), for 30 min at 4 ºC, and centrifuged (15.000x g, 4 ºC, 20 min) to obtain supernatant. Total proteins were quantified in supernatant (PierceTM BCA Protein Assay Kit, ThermoFisher Scientific), and interferon gamma (IFN-γ) (Sigma-Aldrich), IL-1β, IL-6, TNFα, (Elabscience, Houston, TX, USA) and IL-10 (Abcam, Cambridge, MA, USA) were measured by ELISA, according to the manufactures instructions. Results are presented as cytokine concentration per mg of protein.

### Real time PCR

Total ribonucleic acid (RNA) was isolated from frozen brains using TRizol® Reagent according to the manufacturer’s instructions. TNF-α messenger RNA (mRNA) was measured by real time quantitative polymerase chain reaction (qPCR) using GoTaq qPCR Master mix (Promega, Madison, WI, USA). Glyceraldehyde 3-phosphate dehydrogenase (GAPDH) was used as an internal control. qPCR reactions were performed, recorded, and analyzed using the Corbett Research system (Corbett Life Sciences, Sydney, Australia). The conditions for qPCR were as follows: 95 °C for 20 s, 40 cycles of 95°C for 3 s and 60°C for 30 s. Cycle threshold (Ct) values obtained for TNF-α were related to GAPDH (ΔCt) and converted to the linear form using the term 2−ΔΔCt as a value directly proportional to the number of copies of complementary DNA and the initial quantity of mRNA. Primer sequences: TNF-α, F: ATGAGCACAGAAAGCATGATC; R: TACAGGCTTGTCACTCGAATT (275pb) (NM_013693.2); GAPDH, F: GGGCAGCCCAGAACATCAT; R: CCGTTCAGCTCTGGGATGAC (76 bp) (NM_017008.4).

### Flow cytometry

After blood collect, erythrocytes were lysed by using a commercially available hypotonic lysis solution (BD FACSTM, BD Biosciences, San Jose, CA, USA), leukocytes obtained after centrifugation (600x g, 10 mins) were resuspended in Hank’s balanced salt solution (HBSS) (Thermo Fisher Scientific) added 0.1% bovine serum albumin (BSA, Sigma-Aldrich), and incubated with anti-L-selectin fluorescein-conjugated (10 µL/10^6^ cells, Bio-Techne, Minneapolis, MN, USA) or anti-ICAM-1 Alexa Fluor® 488-conjugated monoclonal antibody (1 µg, Santa Cruz Biotechnology, Dallas, TX, USA) for 20 min, at 4ºC. Negative control was not added any antibody. Leukocytes were washed with HBSS plus BSA buffer, fixed in 4% paraformaldehyde solution for 10 min, and fluorescence was measured 12 h later in a flow cytometer (BD Accuri C6, BD Biosciences, San Jose, CA, USA). Ten thousand cells were counted per sample.

### Immunohistochemistry

Brains collected 6 h after induction of sepsis were immersed in a 4% paraformaldehyde solution (Labsynth) for 24 h, following immersion in 30% sucrose solution (Labsynth) for additional 24 h, were embedded in O.C.T freezing medium (Sakura, Alphen aan den Rijn, Holanda) and kept in −80^o^C freezer until use. Eight micrometers sections were obtained in cryostat (Leica, CM1850, Leica Microsystems, Wetzlar, Germany), endogenous peroxidase blocked using 0.3% hydrogen peroxide solution (prepared in metanol) for 10 min, and washed in 1x PBS solution added 0.3% Tween 20 (Sigma-Aldrich). Nonspecific protein-binding sites were blocked using 3% BSA (Sigma-Aldrich) for 30 min, at room temperature. Sections were then incubated with anti-ICAM-1 monoclonal primary antibody (1:1000, vol:vol, Abcam) for 2 h in a humidity chamber. As negative control, no primary antibody was added to some sections. Horseradish peroxidase (HRP)-conjugated secondary antibody (1:1000, vol:vol, Santa Cruz Biotechnology) was added to the sections for 1 h, except for some sections, used as an additional negative control in which no secondary antibody was added (just the primary antibody was used). Labeling was visualized after incubation with a Peroxidase Substrate kit (Vector Laboratories, CA, USA) for 5 to 15 min. Entellan® (Merck, EUA) was used to mount the glass slides, images were obtained using optical microscopy (Leica DMLFS, DFC300 FX, Leica Microsystems), a 20X objective for observation (HCX PL FLUOTAR, Leica Microsystems), a software for capturing (LEICA IM50, Leica Microsystems), and analysis were carried out using the software Image J (Wayne Rasband, National Institute of Health, USA). Results are presented as arbitrary units.

### Western blotting

Frozen brain was powered, homogenized, and supernatant obtained the same way as described in the " Cytokines measurement in brain" Section. Total proteins were quantified in supernatant (PierceTM BCA Protein Assay Kit, ThermoFisher Scientific), 50 µg of proteins were loaded on a 10% polyacrylamide gel (Sigma-Aldrich) and transferred to a polyvinylidene fluoride membrane (PVDF) (GE Healthcare, Little Chalfant, Burckinghamshire, United Kingdom) for 40 min, 80V and 4 °C. Nonspecific binding was blocked using 3% BSA in tween Tris buffered saline (TTBS) buffer (pH 7.6) (LabSynth) for 1.5 h, at room temperature. Membranes were incubated overnight with anti-phosphorylated IκB (phospho-IκB) or anti-IκB primary antibodies (1:350 and 1:1000, vol:vol, respectively, Cell Signaling Technology, MA, USA) at 4 °C. Actin labeling (1:200, vol:vol, Santa Cruz Biotechnology) after membrane stripping (Restore™ Western Blot Stripping Buffer, Thermo Fisher Scientific) was used as loading control and results were related to it. HRP-conjugated secondary antibody (1:2500, vol:vol, Santa Cruz Biotechnology) was put in contact with the membranes for 1.5 h, at room temperature, blotting was visualized after incubation with a quimoluminescence solution (Pierce® ECL Western Blotting Substrate, Thermo Fisher Scientific), and images captured using a luminescence reader device (Carestream Molecular Imaging, Gel Logic 2200 PRO, Carestream Health, NY, EUA). Blotting density was quantified using the software Image J (Wayne Rasband), and expressed as arbitrary units. Protocol for AP-1 and HIF-1α detection is described in the supplemental material [see Additional file 1].

### Survival rate

Mice were daily followed for 10 days to measure the survival rate after induction of sepsis. Cit-AuNP or saline was injected twice, 2 and 24 h after induction of sepsis, the same protocol and dose described in the " Gold nanoparticle treatment" Section. A group of mice was concomitantly IV treated with an antibiotic regimen composed of imipenem and cilastatin (0.3 mg and 0.6 mg per animal, respectively) (Sigma-Aldrich) injected twice a day for three days (through the femoral vein), starting 2 h after induction of sepsis and at 12 h intervals afterwards, to determine whether any drug interaction would occur compared to a group only treated with imipenem and cilastatin. This antibiotic regimen was previously described in [84], and slightly modified. Alternate femoral veins were used every treatment time.

### Statistical analysis

All results are expressed as mean ± standard error of the mean (SEM). To verify whether there was difference between groups, unpaired t-test, analysis of variance (ANOVA) followed by Tukey test, or Log-rank (Mantel-Cox) test were used. All analyses were performed using the Prism 5 software (GraphPad Software, Inc.), and statistical significance was set as P < 0.05.

## Supplementary Information

**Additional file 1: Figure S1.** Effect of 20 nm citrate-covered gold nanoparticles (cit-AuNP) treatment on the activator protein 1 (AP1) and hypoxia inducible factor 1 (HIF-1α) expressions in brain of mice with sepsis. Cit-AuNP or saline was injected intravenously (IV) 2h after induction of sepsis or the sham-operated procedure. Representative blots (upper panel) and quantification (lower panel) of the AP1 (A) and HIF-1α (B) expressions in brain of mice with sepsis or sham-operated previously treated with saline or cit-AuNP. No change in both AP1 and HIF-1a expressions were observed in brain of mice 6h after induction of sepsis, and cit-AuNP treatment did not modify their expression as well. ANOVA followed by Tukey’s test was used for comparison among groups. Four to six animals per group were used. n. s., not significant; a. u., arbitrary units. **Figure S2.** Transmission electron microscopy of brain tissue of mice with sepsis treated or not with 20 nm citrate-covered gold nanoparticles (cit-AuNP). Saline (A) or cit-AuNP (B) was injected intravenously (IV) 2h after induction of sepsis or the sham-operated procedure, and brains were collected 6h after induction. Photos were randomly selected. No cit-AuNP was noted in brain segments of mice with sepsis 4h after cit-AuNP injection. Black squares are magnified in the superior right side of each photo. AT: axon terminal; M: mitochondria; MA: myelinated axon; N: neuron; UA: unmyelinated axon. Bars: 2 μm (left photos) and 500 nm (right photos).

## Data Availability

The datasets used and/or analysed during the current study are available from the corresponding author on reasonable request.

## Abbreviations

ANOVA: Analysis of variance
AP-1: Activator protein 1
a.u.: Arbitrary units
BBB: Blood brain barrier
BSA: Bovine serum albumin
CAMs: Cell adhesion molecules
CD14: Cluster of differentiation 14
Cit-AuNP: Citrate-covered gold nanoparticles
CLP: Cecal ligation and puncture
CNS: Central nervous system
Ct: Cycle threshold
CVO: Circumventricular organs
EB: Evans blue
EEG: Electroencephalogram
ELISA: Enzyme-linked immunosorbent assay
GAPDH: Glyceraldehyde 3-phosphate dehydrogenase
HBSS: Hank’s balanced salt solution
HFD: High fat diet
HIF-1a: Hypoxia inducible factor 1 alpha
HRP: Horseradish peroxidase
HUVEC: Human umbilical vein endothelial cells
IBS: Institute of Biomedical Sciences
ICAM-1: Intercellular cell adhesion molecule 1
IL-1: Interleukin 1
IL-1β: Interleukin 1 beta
IL-10: Interleukin 10
IL-1R: Interleukin 1 receptor
IL-6R: Interleukin 6 receptor
IFN-γ: Interferon gamma
iNOS: Inducible nitric oxide synthase
IP: Intraperitoneal
IκB: Inhibitor of kappa B
IV: Intravenously
LPS: Lipopolysaccharide
mRNA: Messenger ribonucleic acid
NET: Neutrophil extracellular traps
NF-κB: Nuclear factor kappa B
NO: Nitric oxide
n.s.: Not significant
PBS: Phosphate-buffered saline
Phospho-IκB: Phosphorilated inhibitor of kappa B
PMN: Polymorphonuclear
PMSF: Phenylmethylsulfonyl fluoride
PVDF: Polyvinylidene fluoride
qPCR: quantitative polymerase chain reaction
RNA: Ribonucleic acid
ROS: Reactive oxygen species
SAE: Sepsis associated-encephalopathy
SEM: Standard error of the mean
SPF: Specific-pathogen-free
TLR4: Toll-like receptor 4
TNFα: Tumor necrosis factor alpha
TTBS: Tween Tris buffered saline
USP: University of Sao Paulo
