# Supplementary material for: Gold nanoparticles reduce inflammation in cerebral microvessels of mice with sepsis
Source: J Nanobiotechnology. 2021 Feb 19;19:52. doi: 10.1186/s12951-021-00796-6 (PMC7893894; doi:10.1186/s12951-021-00796-6)
Supplement: Supplementary file 1 — Additional file 1: Figure S1. Effect of 20 nm citrate-covered gold nanoparticles (cit-AuNP) treatment on the activator protein 1 (AP1) and hypoxia inducible factor 1 (HIF-1α) expressions in brain of mice with sepsis. Cit-AuNP or saline was injected intravenously (IV) 2h after induction of sepsis or the sham-operated procedure. Representative blots (upper panel) and quantification (lower panel) of the AP1 (A) and HIF-1α (B) expressions in brain of mice with sepsis or sham-operated previously treated with saline or cit-AuNP. No change in both AP1 and HIF-1a expressions were observed in brain of mice 6h after induction of sepsis, and cit-AuNP treatment did not modify their expression as well. ANOVA followed by Tukey’s test was used for comparison among groups. Four to six animals per group were used. n. s., not significant; a. u., arbitrary units. Figure S2. Transmission electron microscopy of brain tissue of mice with sepsis treated or not with 20 nm citrate-covered gold nanoparticles (cit-AuNP). Saline (A) or cit-AuNP (B) was injected intravenously (IV) 2h after induction of sepsis or the sham-operated procedure, and brains were collected 6h after induction. Photos were randomly selected. No cit-AuNP was noted in brain segments of mice with sepsis 4h after cit-AuNP injection. Black squares are magnified in the superior right side of each photo. AT: axon terminal; M: mitochondria; MA: myelinated axon; N: neuron; UA: unmyelinated axon. Bars: 2 μm (left photos) and 500 nm (right photos). [file 12951_2021_796_MOESM1_ESM.pdf]

Supplemental material

Gold nanoparticles reduce inflammation in cerebral microvessels of mice with sepsis

Davide Di Bella; João P. Ferreira; Renee A. Oliveira; Cinthya Echem; Aline Milan; Eliana H. Akamine; Maria H. Carvalho; Stephen F. Rodrigues

Methods

Western blotting

Supernatant from brain homogenates were obtained and procedures to quantify the transcription factors activator protein 1 (AP1) and hypoxia inducible factor 1 (HIF-1α) were performed the same way described in the western blotting section in the main text. Membranes were incubated overnight with anti-AP1 or anti-HIF-1 α primary antibodies (1:1000 and 1:200, vol:vol, respectively) (Cell Signaling Technology, MA, USA, and Abcam, MA, USA, respectively) at 4°C. Actin labeling (1:200, vol:vol, Santa Cruz Biotechnology) after membrane stripping (Restore™ Western Blot Stripping Buffer, Thermo Fisher Scientific) was used as loading control and results were related to it. Membranes were incubated with HRP-conjugated secondary antibody (1:2500, vol:vol, Santa Cruz Biotechnology) for 1.5h, at room temperature, blotting was visualized after incubation with a quimoluminescence solution (Pierce® ECL Western Blotting Substrate, Thermo Fisher Scientific), and images captured using a luminescence reader device (Carestream Molecular Imaging, Gel Logic 2200 PRO, Carestream Health, NY, EUA). Blotting density was quantified using the software Image J (Wayne Rasband), and expressed as arbitrary units.

Transmission electron microscopy

Brain was collected the same way as described in the main text and chemical fixation was performed in 2.0% glutaraldehyde in 0.1 M cacodylate buffer followed by secondary fixation with

osmium tetroxide 1% in H_2O. Specimens were dehydrated in a graded series of ethanol solutions and propylene oxide was used as a transition solvent. Samples were embedded in epoxy resin (Spurr) overnight, transferred and orientated to a silicone mold that was filled with fresh resin and polymerized at 70°C for 24–48 h. Ultrathin sections (90nm) were made in a LEICA Ultramicrotome EM UC6 (Wetzlar, Germany), collected on copper grids and double stained with lead citrate and uranyl acetate. Images were obtained under an JEM1400Plus (Jeol, Japan), operating at 120 kV. Each experimental group was observed in at least four randomly selected fields.

Statistical analysis

All results are expressed as mean ± standard error of the mean (SEM). To verify whether there was difference between groups, ANOVA followed by Tuker test was used. All analyses were performed using the Prism 5 software (GraphPad Software, Inc.), and statistical significance was set as $P < 0.05$.

Results

Transcription factors expression in brain

Sepsis did not change the AP1 and HIF-1 α expression in brain of mice (Fig. S1A and S1B). Cit-AuNP treatment performed 2h after sepsis induction did not change the AP1 and HIF-1α expressions in brain of mice as well (Fig. S1A and S1B).

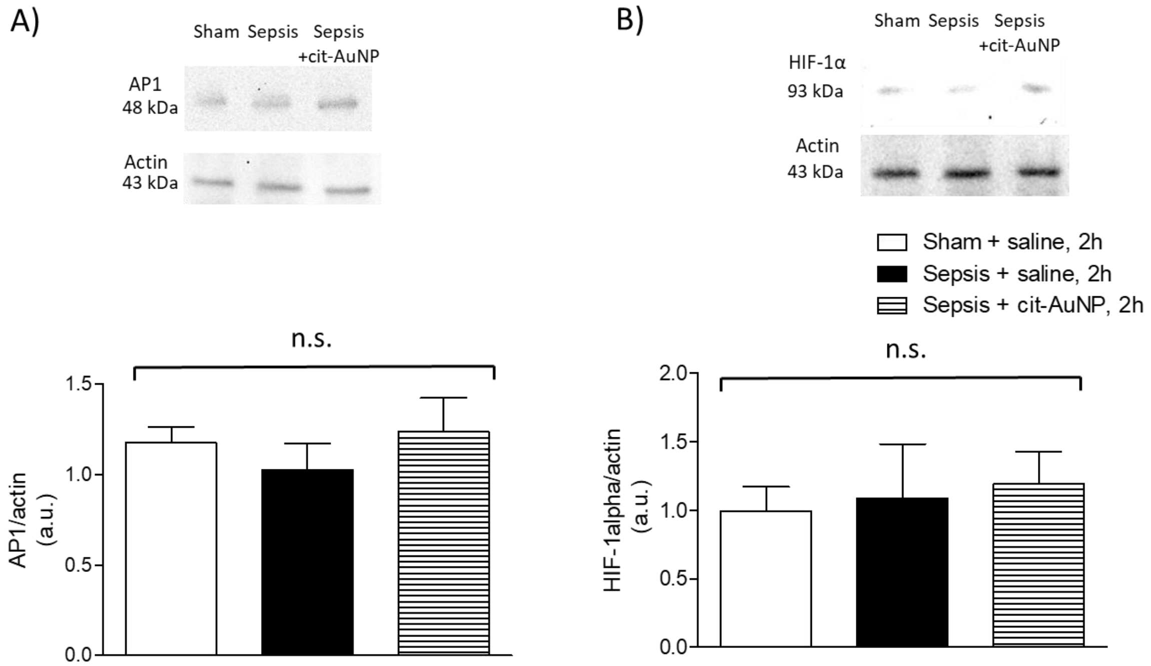

Figure S1. Effect of 20 nm citrate-covered gold nanoparticles (cit-AuNP) treatment on the activator protein 1 (AP1) and hypoxia inducible factor 1 (HIF-1α) expressions in brain of mice with sepsis. Cit-AuNP or saline was injected intravenously (IV) 2h after induction of sepsis or the sham-operated procedure. Representative blots (upper panel) and quantification (lower panel) of the AP1 (A) and HIF-1α (B) expressions in brain of mice with sepsis or sham-operated previously treated with saline or cit-AuNP. No change in both AP1 and HIF-1a expressions were observed in brain of mice 6h after induction of sepsis, and cit-AuNP treatment did not modify their expression as well. ANOVA followed by Tukey's test was used for comparison among groups. Four to six animals per group were used. n. s., not significant; a. u., arbitrary units.

Gold nanoparticles in the cerebral parenchyma

Four hours after cit-AuNP was intravenously injected in mice with sepsis, we could not observe any distinguishable cit-AuNP around the brain parenchyma compared to sham-operated mice treated with saline (Fig. S2A and S2B).

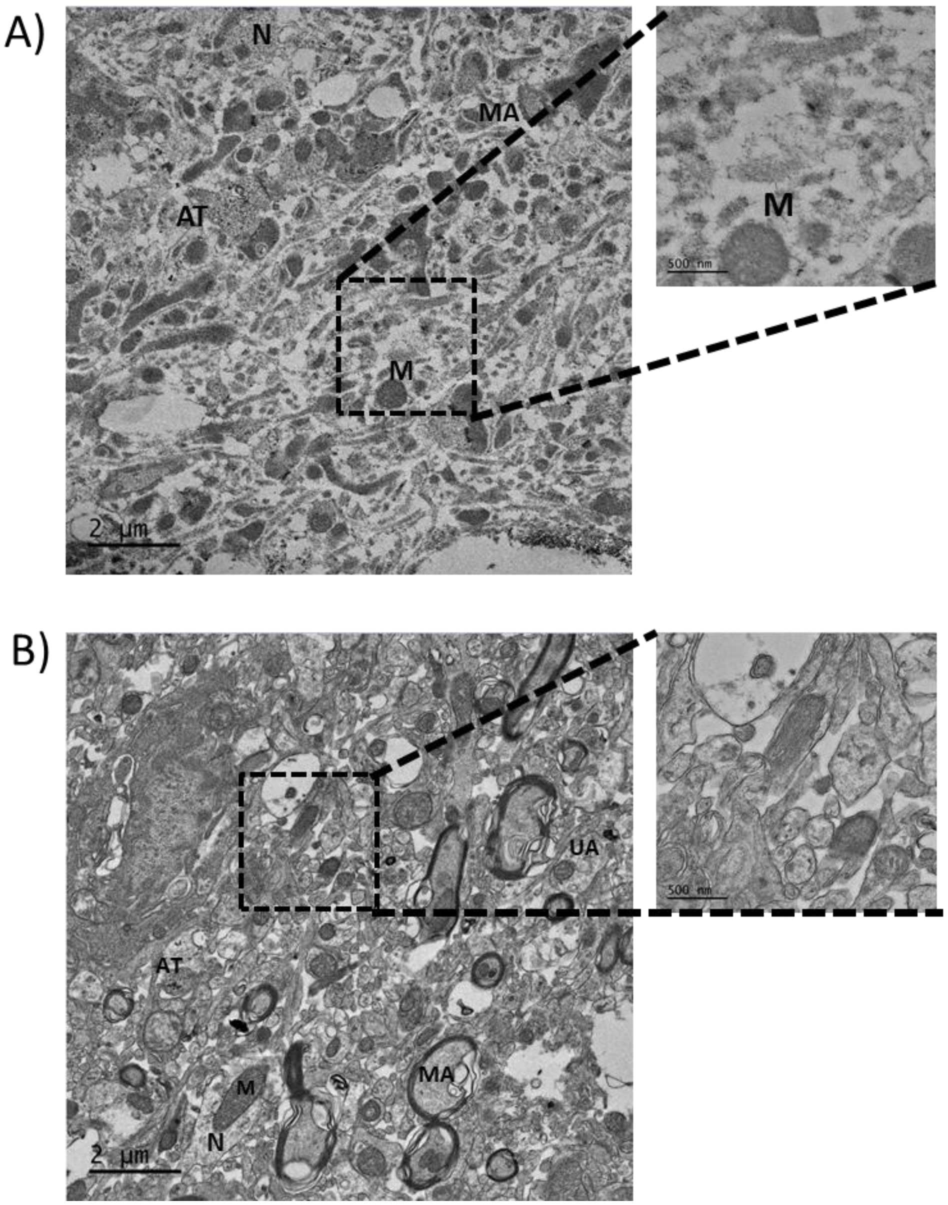

Figure S2. Transmission electron microscopy of brain tissue of mice with sepsis treated or not with 20 nm citrate-covered gold nanoparticles (cit-AuNP). Saline (A) or cit-AuNP (B) was injected intravenously (IV) 2h after induction of sepsis or the sham-operated procedure, and brains were collected 6h after induction. Photos were randomly selected. No cit-AuNP was noted in brain segments of mice with sepsis 4h after cit-AuNP injection. Black squares are magnified in the superior right side of each photo. AT: axon terminal; M: mitochondria; MA: myelinated axon; N: neuron; UA: unmyelinated axon. Bars: 2 µm (left photos) and 500 nm (right photos).
